# Of the Advantages of Local Stimuli, and Particularly Nitrate of Silver, in Ophthalmia

**Published:** 1855-02

**Authors:** 


Of the Advantages of Local Stimuli, and particularly Nitrate of Silver, in Ophthalmia. By Mr. Critchett.—Mr. Critchett entertains the highest opinion of the benefits arising from this kind of treatment in catarrhal and purulent ophthalmia.

Of *catarrhal ophthalmia* he says: "If this affection be correctly diagnosed and attacked early, it may be cured rapidly and almost invariably by local stimuli; and of these, by far the best, according to my experience, is a weak solution of the nitrate of silver, in strength about two grains to the ounce, of distilled water, gradually increased to six grains. If applied in the early stage, however severe and well-marked the disease may be, it will generally subside in a few days. The time required for cure being, according to my observation, pretty accurately measured by the interval between the first development of the disease, and the commencement of the remedy. As, for example, if the treatment be commenced on the third day, it will be well by the sixth day, and so on. I know of nothing that more thoroughly deserves the name of a specific than this. It should be carefully dropped in the eye with a camel's-hair brush or a quill, and may be repeated every three or four hours in severe cases. It causes slight smarting at first, which rapidly subsides, and then the patient feels great relief. The more completely the case is adapted for this treatment, the less pain does it occasion, and the more speedily does it pass off. It is in the epidemic form that the beneficial effect is the most rapid and clearly marked. If there be any unusual complication, as one or more pustules or aphtha, or if there be much constitutional disturbance, the effect is less decided and satisfactory, and it even sometimes fails, and must be discontinued, some astringent, as a solution of alum, being substituted. Some recommend that the caustic should be applied in a much less diluted form. Thus, Mr. Guthrie is, I believe, in the habit of using a strong ointment, containing ten grains of the nitrate of silver to a drachm of lard. As I usually succeed with the milder solution, I cannot speak from personal experience respecting this ointment, but I have no doubt it answers perfectly well, the important point being rather, the suitability of the case than the strength of the stimulus.

"I feel that the profession is much indebted to Mr. Guthrie for bringing prominently before them the value of nitrate of silver in eye disease; at the same time it is to be regretted that this distinguished surgeon, whilst probably instinctively selecting the suitable forms for its application in his own practice, has not very clearly defined the cases to which it should be limited. For we must ever remember that, like other powerful agents, it is equally efficient for good or for evil; whilst it is a remedy of sovereign value in suitable cases, it is most injurious when misapplied, and I have frequently known it to set up a specific inflammation, a sort of nitrate of silver disease, that is most intractable and distressing. The rule I lay down is this, *that in genuine catarrhal disease, it is a specific, and that it is useful in all cases in which the discharge from the conjunctival membrane is of a muco-purulent or purulent character, provided the disease is limited to that membrane, and has not extended to the cornea*

*or other tissues of the eye.* I find that relief ought very speedily to follow the application, and that the pain is slight; if therefore this be not the case, if the symptoms are decidedly aggravated, and the pain increased after a few applications, it is better not to persevere. The case has probably been mistaken, and is unsuited, or some complication has been overlooked; and we must always remember that in using this remedy, if we are not doing good we are doing harm.

"I have dwelt thus minutely, and at some length, upon this plan of treatment, because I believe that there exists in the minds of most medical men, and also in the pages of most ophthalmic works, very vague ideas respecting the use of the nitrate of silver in diseases of the eye, both as regards its value and the cases to which it is applicable. One of the most practical works we have on eye disease—viz., that by the late Mr. Tyrrell,—condemns the use of the nitrate of silver, *in toto;* others seem almost equally indiscriminate in its commendation; it is therefore not to be wondered at that those who have not extensive opportunities of bringing these diverse and contradictory views to the test of experience should acquire confused and erroneous opinions on this truly important practical point.

"In advocating the use of a solution of the nitrate of silver in catarrhal ophthalmia, I do dot wish to limit the treatment to this particular remedy; I am merely desirous of setting forth the stimulating plan as contrasted with the anti-phlogistic, which is strongly advocated by some high authorities, and which I feel satisfied will not control these specific inflammations, of mucous membranes. Other stimuli, particularly alum and the sulphate of copper in substance, may be employed with advantange; but I think, as a general rule, the nitrate of silver is the best; in fact, I have found it too uniformly successful, where the cases have been properly selected, to allow me to doubt its specific power over the disease. The only treatment I adopt in addition to this is, to smear the eyelids at night with spermaceti ointment, to prevent agglutination in the morning. No medicine of any kind is usually required, and the ordinary diet may be continued throughout the treatment of the case."

Mr. Critchett speaks in the same way of purulent ophthalmia:—

"The treatment of these cases is merely local; it is very simple, and the result highly satisfactory. All that is required is the frequent application of some mild astringent or caustic lotion to the surface of the conjunctiva. At the Ophthalmic Hospital we use a solution of alum, from five to ten grains to the ounce; but a weak solution of nitrate of silver answers equally well, and I think acts more rapidly. The essential point is, that whatever is used is well applied to the surface of the membrane. A frequent source of failure is to be traced to the neglect of this measure.

The remedy has been judiciously chosen, but has failed for want of being properly applied. If a lotion is used, it should be frequently injected with a syringe between the lids, so as to wash away the discharge, and get well over the surface of the membrane; if drops are used, the eye should be first carefully cleansed. This plan of treatment, when properly carried out, is almost uniformly successful. Out of many hundred cases that I have seen, I can scarcely recall a single instance where sight has been lost, if the treatment has been commenced sufficiently early in the disease.

"Often as this plan of treatment has been urged, and unanimously as it has been agreed upon by ophthalmic surgeons, simple as it is in its application, and certain in its results, yet painful experience proves that it constantly requires to be reiterated with increased emphasis. It is not unfrequently our painful duty to witness cases of this kind where sight is damaged and even destroyed for lack of a little practical knowledge of this subject on the part of some of my professional brethren. If the despairing aspect and piteous cry of but one poor mother upon whose mind the sad truth suddenly breaks in that her child is hopelessly blind, could image itself to the sight and echo in the ears of those members of our profession, it surely would arouse attention to the importance of devoting a few thoughtful hours and some anxious care to this disease. So strongly have I been impressed upon this subject when I have had a case brought to me hopelessly blind, and have found that it has been under inefficient medical treatment, that I have felt that if it were permitted me to whisper but one short sentence in the ear of every member of our profession that should contain the essence of the greatest good to humanity with which I am acquainted, the one I would select in preference to all others would be—" *Local stimuli should be applied early, often, and thoroughly to the conjunctival surface in purulent ophthalmia of infants.*"—*London Lancet.*

